# Translating Real-World Safety and Implementation Gaps Into a Deployment-Derived AI Readiness Preimplementation Checklist for NHS Health Care Providers: Checklist Development Study

**DOI:** 10.2196/93900

**Published:** 2026-09-28

**Authors:** Adesina Adesuyi, Sid Singh, Vinod Patel, Ponnusamy Saravanan

**Affiliations:** 1 Department of Clinical Informatics, George Eliot Hospital NHS Trust, College Street, Nuneaton, England, CV10 7DJ, United Kingdom, 44 7935112179; 2 Department of Diabetes, Endocrinology and Metabolism, George Eliot Hospital, Nuneaton, United Kingdom; 3 Warwick Academic Health, Warwick Medical School, University of Warwick, Coventry, United Kingdom; 4 Centre for Early Life, University of Warwick, Coventry, United Kingdom; 5 Warwick Centre for Global Health, University of Warwick, Coventry, United Kingdom

**Keywords:** artificial intelligence, AI, clinical decision support, implementation science, clinical informatics, digital health governance, NHS, patient safety, AI readiness assessment

## Abstract

**Background:**

AI systems are increasingly deployed across National Health Service (NHS) services, yet safety and implementation challenges may only become apparent after clinical go-live. Existing governance and implementation frameworks provide valuable high-level guidance, but health care provider organizations still require practical, auditable tools to support preimplementation decision-making.

**Objective:**

This study aimed to develop a deployment-derived AI readiness checklist and assess its early feasibility, face validity, and content validity within the originating NHS Trust context.

**Methods:**

We conducted a pragmatic checklist development study with retrospective structured application in a UK NHS district general hospital (George Eliot Hospital NHS Trust). The SID & ADE AI Pre-Implementation Checklist was developed from empirical learning across trust AI deployment activity, primarily an AI fracture detection system and an AI-supported prostate magnetic resonance imaging pathway. Evidence sources included a clinico-AI discordance study, the Quality, Service Improvement and Redesign program using plan-do-study-act cycles, and governance artifacts from AI deployment activities. Safety, governance, operational, workforce, information governance, procurement, and monitoring gaps were translated into auditable preimplementation requirements. The checklist was retrospectively applied to the same deployments from which it was derived to assess readiness completeness and demonstrate face and content validity within the originating context. This design was not intended to establish independent construct or predictive validity.

**Results:**

The checklist comprises 8 domains: use-case definition; clinical safety and accountability; local validation and performance; workforce readiness and human factors; operational and technical integration; information governance and ethics; procurement, liability, and financial risk; and monitoring, evaluation, and stop rules. Retrospective application demonstrated variability in readiness completeness across domains, with recurrent gaps in workforce readiness, local validation, and monitoring. The process highlighted areas where structured pre–go-live deliberation may have prompted earlier remediation and clearer governance action.

**Conclusions:**

The SID & ADE AI Pre-Implementation Checklist translates real-world AI deployment learning into a practical preimplementation deliberation tool. Current evidence supports face and content validity within the originating trust context, but independent prospective validation is required before claims of predictive validity, generalizability, or quantitative go-live thresholds can be made.

## Introduction

AI and machine-learning systems are increasingly deployed across health care to support diagnostic interpretation, clinical decision support, workflow optimization, and service efficiency [1,2]. Despite this rapid expansion, real-world implementation has frequently proven challenging, with many failures attributable not to algorithmic performance but to sociotechnical, organizational, and governance factors [3,4].

For the purpose of this study, “operational evaluation” refers to assessment of an AI system within a live or near-live clinical workflow, including interaction with clinical users, integration into existing care pathways, and influence on real-world decision-making. This differs from offline model validation, in which an algorithm is tested retrospectively on static datasets without integration into clinical workflow or user interaction. For example, testing a fracture detection model on historical radiographs represents offline validation, whereas use of the same model within a picture archiving and communication system (PACS)–integrated reporting or emergency care workflow with clinician review represents operational evaluation.

Clinical AI introduces new categories of patient safety risk. Automation bias, defined as overreliance on algorithmic outputs, has been consistently documented in clinical decision support systems and may be exacerbated in high-throughput or time-pressured environments [5,6]. In parallel, AI systems are vulnerable to dataset shift and performance degradation over time, underscoring the need for life cycle governance, continuous monitoring, and postdeployment quality improvement [7-9].

International bodies have emphasized ethical principles, transparency, human oversight, and accountability for AI in health [10]. In England, these principles are operationalized through digital clinical safety standards DCB0129 [11] and DCB0160 [12], which require structured hazard identification, risk mitigation, and clinical safety cases for health IT deployments [13]. However, while such frameworks clarify what good governance looks like, they offer limited support for operational go-live decisions at the health care provider level.

Implementation science frameworks such as the nonadoption, abandonment, scale-up, spread, and sustainability framework (NASSS) and the Consolidated Framework for Implementation Research (CFIR) provide valuable lenses for understanding adoption, nonadoption, and sustainability [14,15]. Nevertheless, these frameworks are descriptive rather than prescriptive and are not designed as auditable decision tools. This study addresses this gap by deriving a preimplementation AI readiness checklist directly from real-world deployment experience and providing early feasibility and face validity assessment through retrospective application.

## Methods

### Study Design

We conducted a pragmatic checklist development study with retrospective structured application, aligned with implementation science and quality improvement principles [15,16]. The study was designed to generate a deployment-derived AI readiness tool, named the SID & ADE AI Pre-Implementation Checklist (Multimedia Appendix 1), and to assess its feasibility, face validity, and content validity within the originating George Eliot Hospital NHS Trust context. It was not designed to establish independent construct validity, predictive validity, or generalizability.

#### Operational Evaluation and Maturity Framing

Operational evaluation was defined as assessment of AI in live or near-live clinical workflow, including user interaction, pathway integration, and influence on operational or clinical decision-making. This was distinguished from offline model validation, in which algorithmic performance is tested retrospectively without clinical workflow integration. A formal DECIDE-AI (Developmental and Exploratory Clinical Investigations of Decision support systems driven by Artificial Intelligence) [13] maturity classification was not part of the original study design. However, DECIDE-AI–informed terminology was used descriptively to distinguish between offline validation, early clinical evaluation, operational deployment, and postdeployment monitoring. This descriptive maturity framing was intended to improve transparency and does not imply formal DECIDE-AI compliance.

#### Data Sources

Checklist development drew on empirical learning from trust AI deployment activity, primarily (1) a clinico-AI discordance study conducted during deployment of an AI fracture detection system and (2) a Quality, Service Improvement and Redesign (QSIR) program [17] using plan-do-study-act (PDSA) cycles [16] during fracture AI implementation. Additional contextual learning was drawn from governance and implementation artifacts associated with an AI-supported prostate magnetic resonance imaging (MRI) pathway. Subsequent early internal use of the checklist in other trust AI projects, including chest pathology AI and a research-linked lung imaging AI collaboration, informed feasibility reflections but was not treated as independent validation.

### Checklist Development Process

Checklist items were generated through structured extraction of recurring safety, governance, operational, workforce, information governance, procurement, and monitoring gaps identified during trust AI deployment activity. Initial items were grouped into domains and refined iteratively through multidisciplinary discussion involving clinical informatics leadership, clinical safety, nursing informatics, digital nursing, radiology, clinicians, information governance, business intelligence, data science, AI developers, research and development, and audit/quality improvement stakeholders.

Items were retained where they represented a recurrent deployment requirement, a safety-critical precondition, or a governance artifact required for safe implementation. Items were revised or merged where duplication was identified, and wording was refined to ensure that each requirement could be supported by evidence or comments. The final checklist comprised 8 domains: use-case definition; clinical safety and accountability; local validation and performance; workforce readiness and human factors; operational and technical integration; information governance and ethics; procurement, liability, and financial risk; and monitoring, evaluation, and stop rules.

No formal Delphi consensus, content validity index, item-reduction statistics, or external independent expert panel was used. Framework mapping was performed post hoc by the authoring team to assess conceptual alignment with NASSS, CFIR, World Health Organization (WHO) AI governance guidance, and National Health Service (NHS) digital clinical safety standards. This approach prioritizes ecological validity and operational relevance but limits claims about formal content validity and generalizability [10,13-15].

### Reporting Framework

The study was reported with reference to the Standards for Reporting Implementation Studies (StaRI), as the work concerns implementation processes and adoption in a real health care setting [18]. DECIDE-AI was considered relevant for maturity framing because it addresses early-stage clinical evaluation of AI decision-support systems, but the present study does not evaluate AI model performance and therefore does not fully follow DECIDE-AI. COSMIN (Consensus-based Standards for the selection of health Measurement Instruments) guidance was considered but not applied because the SID & ADE checklist is not a patient-reported outcome measure or psychometric measurement instrument [19,20].

#### Retrospective Readiness Gap Audit

The checklist was retrospectively applied to the fracture AI and prostate MRI AI deployments. Each item was rated as present, partially present, or absent at the time of initial go-live or pilot initiation. Present items scored 1, partially present items scored 0.5, and absent items scored 0 for descriptive completeness calculations. Domain completeness was calculated as the sum of item scores divided by the total number of items in that domain, expressed as a percentage. These percentages were used descriptively and were not interpreted as validated go-live thresholds.

Ratings were undertaken by members of the trust clinical AI and governance team familiar with the deployments. Ratings were discussed by multidisciplinary stakeholders, but no blinded independent rating process was used, and no interrater reliability statistic was calculated. Therefore, the audit should be interpreted as structured reflective assessment rather than independent objective measurement.

#### Checklist Administration and Decision-Making

The checklist is intended to be completed by the clinical directorate lead or nominated clinical deployment lead for the pathway in which the AI system will be used. Completion should involve multidisciplinary input from the clinical safety officer, chief clinical information officer or clinical informatics lead, chief nursing information officer or nursing informatics representative where relevant, information governance, digital/IT, business intelligence, and research/audit. The checklist is not currently a quantitative scoring instrument. It is intended to support structured multidisciplinary deliberation. Final go-live decisions should be made through existing trust governance structures, with unresolved disagreements escalated to clinical safety or digital governance groups.

### Ethical Considerations

This work was conducted as a service evaluation and implementation audit of AI deployment processes. No patient-level data were analyzed for the checklist-development component. Staff participation in reflective assessment was voluntary, and responses were anonymized and reported at aggregate level [10,13].

### Retrospective Readiness Gap Audit

Each checklist item was retrospectively assessed as present, partially present, or absent at the time of initial go-live or pilot initiation. Where items were unmet, subsequent implementation challenges were documented, including escalation ambiguity, training, gaps, workflow disruption, and reactive monitoring. Readiness completeness by domain for each deployment is summarized in Table 1.

**Table 1.** Translation of deployment-derived evidence into SID & ADE AI Pre-Implementation Checklist requirements. Framework alignment was conducted post hoc by the authoring team and was used to assess conceptual fit rather than to claim formal external validation.

Evidence source	Observed gap or risk during deployment	Risk category	Resulting checklist requirement	Framework alignment
Clinico-AI discordance (fracture AI)	Unclear escalation for AI-clinician disagreement	Clinical safety	Defined escalation pathway for discordant AI outputs	NASSS^a^ (organization); DCB0160
Clinico-AI discordance (fracture AI)	Automation bias in borderline cases	Human factors	Training on AI limitations and override expectations	CFIR^b^ (individuals); WHO^c^
QSIR^d^/PDSA^e^ cycles (fracture AI)	Workflow disruption at early go-live	Operational	End-to-end workflow integration tested before go-live	NASSS (technology/organization)
QSIR/PDSA cycles (fracture AI)	Variable staff confidence across shifts	Workforce readiness	Role-specific training completed predeployment	CFIR
QSIR/PDSA cycles (fracture AI)	Reactive issue detection	Monitoring	Defined monitoring metrics and feedback loops	WHO; NASSS
MRI^f^ prostate AI retrospective review	No local performance assurance at go-live	Validation	Local retrospective validation completed	WHO; NASSS
MRI prostate AI governance review	Ambiguity of AI role (assistive vs advisory)	Adoption	Explicit statement of AI role and intended use	NASSS
Cross-deployment learning	Absence of stop/suspend criteria	Safety governance	Predefined stop rules and suspension triggers	DCB0160

a NASSS: nonadoption, abandonment, scale-up, spread, and sustainability framework.

b CFIR: Consolidated Framework for Implementation Research.

c WHO: World Health Organization.

d QSIR: Quality, Service Improvement and Redesign.

e PDSA: plan-do-study-act.

f MRI: magnetic resonance imaging.

## Results

### Deployment Evidence and Maturity Framing

The 2 principal source deployments differed in clinical pathway, operational maturity, and role in checklist development. The fracture AI deployment represented a more advanced operational implementation with postdeployment discordance monitoring and QSIR/PDSA learning, whereas the prostate MRI AI pathway represented an earlier-stage pathway evaluation with stronger emphasis on governance preparation, retrospective local validation planning, and pathway integration [5,7]. Table 2 summarizes the evaluation environments and implementation maturity of the source deployments.

**Table 2.** Deployment characteristics and maturity.

Deployment	Clinical setting	AI function	Evaluation environment	Operational maturity	Role in this study
AI fracture detection	Emergency/radiology pathway	Diagnostic support for fracture detection	PACS-integrated^a^ clinical workflow with clinician review	Operational deployment with postdeployment monitoring	Primary source of discordance, safety, workflow, and monitoring learning
AI-supported prostate MRI^b^ pathway	Radiology/urology pathway	Imaging support and pathway decision support	Retrospective pathway evaluation and governance preparation	Early operational readiness pilot phase	Source of governance, validation, pathway integration, and role-clarity learning
Qure/chest pathology AI	Radiology/chest imaging pathway	Diagnostic support for chest pathology	Internal trust deployment and governance use	Early internal feasibility use	Informed feasibility reflections only; not treated as independent validation
Research-linked lung imaging AI collaboration	Research/clinical imaging interface	Radiomics and high-precision machine learning support	Research-linked implementation planning	Predeployment research-to-clinical transition	Informed feasibility reflections only; not treated as independent validation

a PACS: picture archiving and communication system.

b MRI: magnetic resonance imaging.

### Translation of Deployment Evidence Into Checklist Requirements

Deployment-derived evidence was translated into auditable readiness requirements. Recurrent themes included unclear escalation routes for AI-clinician disagreement, automation bias risk, variable staff confidence, workflow disruption, incomplete local validation, and limited prespecified monitoring or stop criteria. These were translated into checklist requirements covering escalation pathways, human oversight, role-specific training, workflow testing, local validation, and monitoring plans. Table 1 summarizes how observed deployment gaps informed checklist items and how these items aligned conceptually with implementation and governance frameworks.

### Retrospective Readiness Completeness

Retrospective application of the checklist demonstrated variability in readiness completeness across domains. Information governance and procurement-related domains had relatively higher completeness, whereas workforce readiness, local validation, and monitoring/stop-rule domains were less complete at the point of go-live or pilot initiation [4,8]. Domain completeness values are reported descriptively in Table 3 with denominators to enable reconstruction of percentages. These values should not be interpreted as validated thresholds or objective external measures because formal interrater reliability was not assessed.

**Table 3.** Retrospective readiness completeness at go-live across 2 AI deployments. Item-level scoring was assigned as present=1, partially present=0.5, and absent=0. Domain completeness values represent the sum of item scores divided by the total number of items within that domain and are expressed descriptively as percentages. These values are intended to support reflective comparison rather than to function as validated quantitative thresholds for deployment decisions.

Checklist domain	Number of items	Fracture AI score/maximum (%)	Prostate MRI^a^ AI score/maximum (%)	Key gap at go-live or pilot initiation	Observed consequence or remediation area
Use-case definition	7	6.0/7.0 (86)	5.0/7.0 (71)	AI role clarity in prostate MRI	Early variation in interpretation of intended use
Clinical safety and accountability	7	6.0/7.0 (86)	5.0/7.0 (71)	Escalation clarity	Escalation pathways refined after initial implementation
Local validation and performance	6	4.0/6.0 (67)	3.0/6.0 (50)	Local/subgroup assurance	Reduced confidence in edge cases and need for further validation
Workforce readiness and human factors	5	3.0/5.0 (60)	2.0/5.0 (40)	Training coverage and automation bias preparation	Uneven adoption and variable confidence
Operational and technical integration	5	4.0/5.0 (80)	3.0/5.0 (60)	Fallback workflow clarity	Temporary workflow disruption or need for pathway adjustment
Information governance and ethics	6	6.0/6.0 (100)	6.0/6.0 (100)	None identified	No major information governance remediation identified
Procurement, liability, and financial risk	5	4.0/5.0 (80)	4.0/5.0 (80)	Exit/decommissioning planning	Future contract and decommissioning planning required
Monitoring, evaluation, and stop rules	5	2.0/5.0 (40)	1.0/5.0 (20)	Drift monitoring and stop/suspend criteria	Monitoring arrangements developed reactively

a MRI: magnetic resonance imaging.

### Face and Content Validity Within Originating Context

The retrospective application demonstrated that the checklist captured issues recognizable to stakeholders involved in the deployments and reflected domains considered important for safe preimplementation deliberation. This supports face and content validity within the originating trust context. However, because the checklist was derived from the same deployments to which it was applied, this analysis does not establish independent construct validity, predictive validity, or generalizability.

### Early Feasibility Observations

Early internal use suggested that the checklist can be incorporated into existing governance discussions, particularly in which a clinical directorate lead is responsible for coordinating completion and evidence gathering. Formal usability testing, completion-time measurement, and acceptability assessment were not undertaken. These will be incorporated into the next phase of prospective validation.

## Discussion

### Principal Findings

This study developed the SID & ADE AI Pre-Implementation Checklist (Multimedia Appendix 1) as a deployment-derived tool to support structured pre–go-live deliberation for AI systems in an NHS health care provider organization. The checklist was informed by real-world safety, governance, workforce, operational, and monitoring gaps observed across trust AI deployment activity. Retrospective application showed that the tool captured issues recognizable to implementation stakeholders and provided a structured way to organize preimplementation readiness discussions. However, because the checklist was derived from the same deployments to which it was retrospectively applied, the findings demonstrate face and content validity within the originating context only, not independent construct or predictive validity.

### Relationship to Existing Frameworks and Tools

The SID & ADE checklist differs from higher-level frameworks such as NASSS and CFIR, which help explain adoption complexity but do not function as local go-live decision tools. NASSS is valuable for understanding nonadoption, abandonment, scale-up, spread, and sustainability, while CFIR provides a taxonomy of implementation determinants [14,15]. The SID & ADE checklist translates these broad implementation concepts into auditable preimplementation requirements that can be reviewed by clinical, informatics, and governance teams before deployment.

The checklist also differs from DECIDE-AI, which provides reporting guidance for early-stage clinical evaluation of AI decision-support systems and is particularly useful for transparency and reproducibility in AI evaluation studies [13]. The SID & ADE checklist is not a reporting guideline for AI model evaluation; rather, it is a local governance and implementation tool designed to help health care provider organizations decide whether key preconditions for safe deployment have been met. Similarly, WHO guidance and NHS DCB0129/DCB0160 standards set important ethical and clinical safety expectations, but they do not in themselves provide a single pathway-level checklist that integrates clinical safety, workforce readiness, information governance, local validation, and monitoring into one pre–go-live deliberation process [21].

### Practical Implications for NHS AI Deployment

The practical contribution of the checklist is its ability to make existing assurance requirements visible and actionable before go-live. In NHS organizations, AI deployment commonly involves multiple teams, including clinical directorates, radiology or specialty services, clinical safety, information governance, digital/IT, business intelligence, procurement, research and development, and suppliers. Without a shared preimplementation artifact, gaps can remain distributed across separate workstreams and become visible only after deployment. The checklist is intended to support clinical ownership by placing the directorate or pathway lead at the center of completion, with sign-off through clinical safety, clinical informatics, information governance, and digital executive structures.

The tool may be particularly useful for identifying safety-critical gaps before implementation, such as absent escalation pathways, incomplete local validation, unclear human oversight, insufficient staff training, and lack of stop/suspend criteria. These are not simply administrative requirements; they are the mechanisms by which AI-related risks are translated into controllable governance actions.

### Usability and Feasibility

The checklist was designed for use within routine governance meetings rather than as a separate research exercise. The expected administration route is completion by the clinical directorate lead or nominated deployment lead, with multidisciplinary input from the clinical safety officer, chief clinical information officer or informatics lead, information governance, digital/IT, business intelligence, research and development/audit, and supplier representatives where applicable. The completed checklist should then support sign-off through the clinical safety officer, chief clinical information officer/clinical informatics, information governance, and digital executive approval route.

Formal usability testing was not undertaken in this phase, and completion time was not measured systematically. Early trust use suggests that the checklist is feasible within existing governance workflows, but this remains an informal observation. Future prospective work should measure time to complete, perceived burden, clarity of items, number of actions generated, and acceptability among frontline clinical teams and governance stakeholders.

### Checklist Fatigue and Implementation Burden

A potential risk is that the checklist could be perceived as another bureaucratic layer in an already demanding NHS governance environment. This is a legitimate concern, particularly in underresourced health care provider organizations. The checklist should therefore not duplicate data protection impact assessment, Digital Technology Assessment Criteria [22], or DCB0129/DCB0160 [11,12] documentation. Its purpose is to signpost, integrate, and make visible the evidence that those processes generate. Future versions may require digital integration, shortened screening versions for low-risk tools, and modular sections tailored to AI type and clinical risk.

### Limitations

The most important limitation is circularity. The checklist was developed from gaps observed in the same deployments to which it was retrospectively applied. Therefore, alignment between checklist items and observed gaps is expected and cannot establish independent validity. The study also used a single NHS trust context, and the principal source deployments were radiology adjacent. Generalizability to generative AI, natural language processing, ambient documentation, patient-facing AI, predictive analytics, other specialties, or other health care systems remains unproven.

A second limitation is the absence of formal instrument-development methods. No Delphi process, content validity index, independent external panel, item reduction statistics, or psychometric testing was performed. Framework mapping was conducted post hoc by the authoring team. These limitations reduce confidence in formal content validity and may introduce developer bias.

A third limitation is the absence of formal interrater reliability assessment. Retrospective ratings were undertaken through structured discussion by stakeholders familiar with the deployments, but no independent blinded raters were used, and no kappa or intraclass correlation statistic was calculated. The completeness percentages should therefore be interpreted as descriptive reflective ratings rather than objective validated measures.

### Future Work

The next phase will prospectively evaluate the checklist across independent AI deployments and across the wider Foundation Group, which includes 3 other NHS trusts. Also, the involvement of other NHS sites existing outside of George Eliot Hospital Foundation Group, such as Nottingham University Hospital NHS Trust, is now in the preliminary phase. This will ensure multiple NHS sites, independent raters, interrater reliability assessment, formal acceptability and usability testing, and predefined evaluation of whether checklist-identified gaps predict subsequent implementation issues. A Delphi or modified consensus process could be used to refine items, define safety-critical “red flag” requirements, and explore whether any domain-level or overall thresholds are appropriate. Until such validation is completed, the checklist should be used as a structured deliberation framework rather than a quantitative go-live scoring instrument.

Prospective validation across the wider Foundation Group and collaborating NHS sites will involve checklist completion and scoring primarily by local deployment and governance teams independent of the original checklist development team. These are expected to include clinical directorate leads, clinical safety officers, chief clinical information officer/chief nursing information officer representatives, information governance, digital/IT, and local implementation stakeholders responsible for the relevant AI deployment pathway. The principal developer may contribute to methodological coordination and implementation support but will not serve as the primary independent rater for prospective deployment assessments. Interrater reliability analysis will therefore be conducted across independent site-based raters.

### Conclusions

The SID & ADE AI Pre-Implementation Checklist translates real-world deployment learning into a structured pre–go-live deliberation tool. The current study supports face and content validity within the originating trust context but does not establish independent validation. The checklist’s potential value lies in making AI readiness visible, auditable, and actionable before deployment. Prospective multisite validation is required to determine its reliability, usability, generalizability, and impact on AI deployment safety.

## Supplementary material

10.2196/93900 Multimedia Appendix 1 SID & ADE AI Pre-Implementation checklist.

## Abbreviations

CFIR: Consolidated Framework for Implementation Research
COSMIN: Consensus-based Standards for the selection of health Measurement Instruments
DECIDE-AI: Developmental and Exploratory Clinical Investigations of Decision support systems driven by Artificial Intelligence
MRI: magnetic resonance imaging
NASSS: nonadoption, abandonment, scale-up, spread, and sustainability framework
NHS: National Health Service
PACS: picture archiving and communication system
PDSA: plan-do-study-act
QSIR: Quality, Service Improvement and Redesign
StaRI: Standards for Reporting Implementation Studies
WHO: World Health Organization
