# Supplementary material for: Translating Real-World Safety and Implementation Gaps Into a Deployment-Derived AI Readiness Preimplementation Checklist for NHS Health Care Providers: Checklist Development Study
Source: JMIR AI. 2026 Sep 28;5:e93900. doi: 10.2196/93900 (PMC13618789; doi:10.2196/93900)
Supplement: Supplementary file 1 [file ai-v5-e93900-s001.docx]

SID & ADE – AI PRE-IMPLEMENTATION CHECKLIST

George Eliot Hospital NHS Trust

# Purpose

To provide a standardised, auditable, and scientifically grounded pre-implementation assurance checklist for determining whether an AI solution is ready to go live at George Eliot Hospital NHS Trust.

Scope

This checklist was developed within a single NHS district general hospital and was initially informed by radiology-adjacent AI deployments, including fracture detection and prostate MRI pathway implementation. It is intended for use as a structured pre-implementation deliberation tool for AI deployments within NHS provider organisations.

Although the checklist domains may be conceptually relevant to other AI types, including generative AI, NLP, ambient documentation, predictive analytics and patient-facing tools, applicability beyond the originating Trust and beyond radiology-adjacent AI remains unproven. Use in other AI modalities, specialties or organisations should therefore be treated as prospective adaptation and validation rather than established generalisability.

# Instructions

For each item, tick the relevant box and add comments/evidence where applicable. Evidence may include documents, links, approvals, audit results, or references.

## **SECTION A: PROJECT & USE-CASE DEFINITION**

| **Item** | **Requirement** | **Yes \| No** | **Comments / Evidence** |
| --- | --- | --- | --- |
| A1 | Project name, supplier, version identified |  |  |
| A2 | Clear clinical / operational problem defined (not technology-led) |  |  |
| A3 | Intended use explicitly stated (who, when, where, why) |  |  |
| A4 | AI role defined (assistive / advisory / triage / automation) |  |  |
| A5 | Non-AI alternatives considered and compared |  |  |
| A6 | Target population and pathway specified |  |  |
| A7 | Expected benefits defined and measurable |  |  |

## **SECTION B: CLINICAL SAFETY & ACCOUNTABILITY**

| **Item** | **Requirement** | **Yes \| No** | **Comments / Evidence** |
| --- | --- | --- | --- |
| B1 | Named Clinical Safety Officer (CSO) assigned |  |  |
| B2 | DCB 0129 supplier safety evidence reviewed |  |  |
| B3 | DCB 0160 local deployment safety case initiated |  |  |
| B4 | Hazard log created (pre-deployment) |  |  |
| B5 | Known failure modes identified (FP, FN, drift) |  |  |
| B6 | Clear escalation and incident reporting pathway defined |  |  |
| B7 | Human oversight and clinical override preserved |  |  |

## **SECTION C: DATA, PERFORMANCE & LOCAL VALIDATION**

| **Item** | **Requirement** | **Yes \| No** | **Comments / Evidence** |
| --- | --- | --- | --- |
| C1 | Training data characteristics disclosed by supplier |  |  |
| C2 | Relevance to GEH population assessed |  |  |
| C3 | Local retrospective validation completed (where applicable) |  |  |
| C4 | Performance thresholds agreed for go-live |  |  |
| C5 | Subgroup performance and bias risks considered |  |  |
| C6 | Explainability and interpretability acceptable to users |  |  |

## **SECTION D: WORKFORCE READINESS & HUMAN FACTORS**

| **Item** | **Requirement** | **Yes \| No** | **Comments / Evidence** |
| --- | --- | --- | --- |
| D1 | Affected staff groups identified |  |  |
| D2 | Role-specific AI training delivered or planned |  |  |
| D3 | AI limitations and failure modes explicitly taught |  |  |
| D4 | Automation bias risk acknowledged and mitigated |  |  |
| D5 | Staff acceptability assessed |  |  |

## **SECTION E: OPERATIONAL & TECHNICAL INTEGRATION**

| **Item** | **Requirement** | **Yes \| No** | **Comments / Evidence** |
| --- | --- | --- | --- |
| E1 | Integration with EPR / PACS / workflow tested |  |  |
| E2 | Infrastructure capacity confirmed |  |  |
| E3 | Downtime and fallback processes defined |  |  |
| E4 | Supplier support and response arrangements agreed |  |  |
| E5 | Business continuity and dependency risks assessed |  |  |

## **SECTION F: INFORMATION GOVERNANCE & ETHICS**

| **Item** | **Requirement** | **Yes \| No** | **Comments / Evidence** |
| --- | --- | --- | --- |
| F1 | DPIA completed and approved |  |  |
| F2 | Data flows mapped end-to-end |  |  |
| F3 | Lawful basis for data use confirmed |  |  |
| F4 | Patient transparency materials prepared (if applicable) |  |  |
| F5 | Secondary data use governed |  |  |
| F6 | Ethical trade-offs documented |  |  |

## **SECTION G: PROCUREMENT, LIABILITY & FINANCIAL RISK**

| **Item** | **Requirement** | **Yes \| No** | **Comments / Evidence** |
| --- | --- | --- | --- |
| G1 | Medical device classification confirmed |  |  |
| G2 | DTAC alignment reviewed |  |  |
| G3 | Liability allocation contractually explicit |  |  |
| G4 | Cost model understood (licence, scaling, exit) |  |  |
| G5 | Exit / withdrawal strategy defined |  |  |

## **SECTION H: MONITORING, EVALUATION & STOP RULES**

| **Item** | **Requirement** | **Yes \| No** | **Comments / Evidence** |
| --- | --- | --- | --- |
| H1 | KPIs defined (clinical, safety, operational) |  |  |
| H2 | Post-deployment audit plan agreed |  |  |
| H3 | Performance drift monitoring planned |  |  |
| H4 | User feedback mechanism established |  |  |
| H5 | Explicit stop / suspend criteria defined |  |  |

# SCIENTIFIC & IMPLEMENTATION FRAMEWORKS USED

## Primary framework

NASSS Framework (Non-Adoption, Abandonment, Scale-up, Spread, Sustainability) – explains why AI succeeds or fails over time in real clinical settings.

## Secondary frameworks

- CFIR – determinants of implementation success
- WHO AI lifecycle governance – ethics, safety, post-market surveillance
- NHS DCB 0129 / 0160 – clinical risk management standards

# Positioning Statement

This checklist does not underestimate earlier frameworks or existing work; rather, it institutionalises them. Other documents explain what AI readiness is. The SID & ADE AI Pre-Implementation Checklist determines whether an AI solution should actually go live.

# PRE-IMPLEMENTATION DECISION

| Decision | Tick |
| --- | --- |
| Ready to deploy | ☐ |
| Deploy with conditions | ☐ |
| Not ready – remediation required | ☐ |

# SIGN-OFF

| **Role** | **Name** | **Signature** | **Date** |
| --- | --- | --- | --- |
| Clinical Lead |  |  |  |
| Clinical Safety Officer |  |  |  |
| CCIO |  |  |  |
| Info. Governance Lead |  |  |  |
| Executive Sponsor |  |  |  |
